# Supplementary material for: The Expanded SWEET Gene Family Following Whole Genome Triplication in Brassica rapa
Source: Genes (Basel). 2019 Sep 18;10(9):722. doi: 10.3390/genes10090722 (PMC6771021; doi:10.3390/genes10090722)
Supplement: Supplementary file 1 [file genes-10-00722-s001.zip › Supplementary files/Figure S2.pdf]

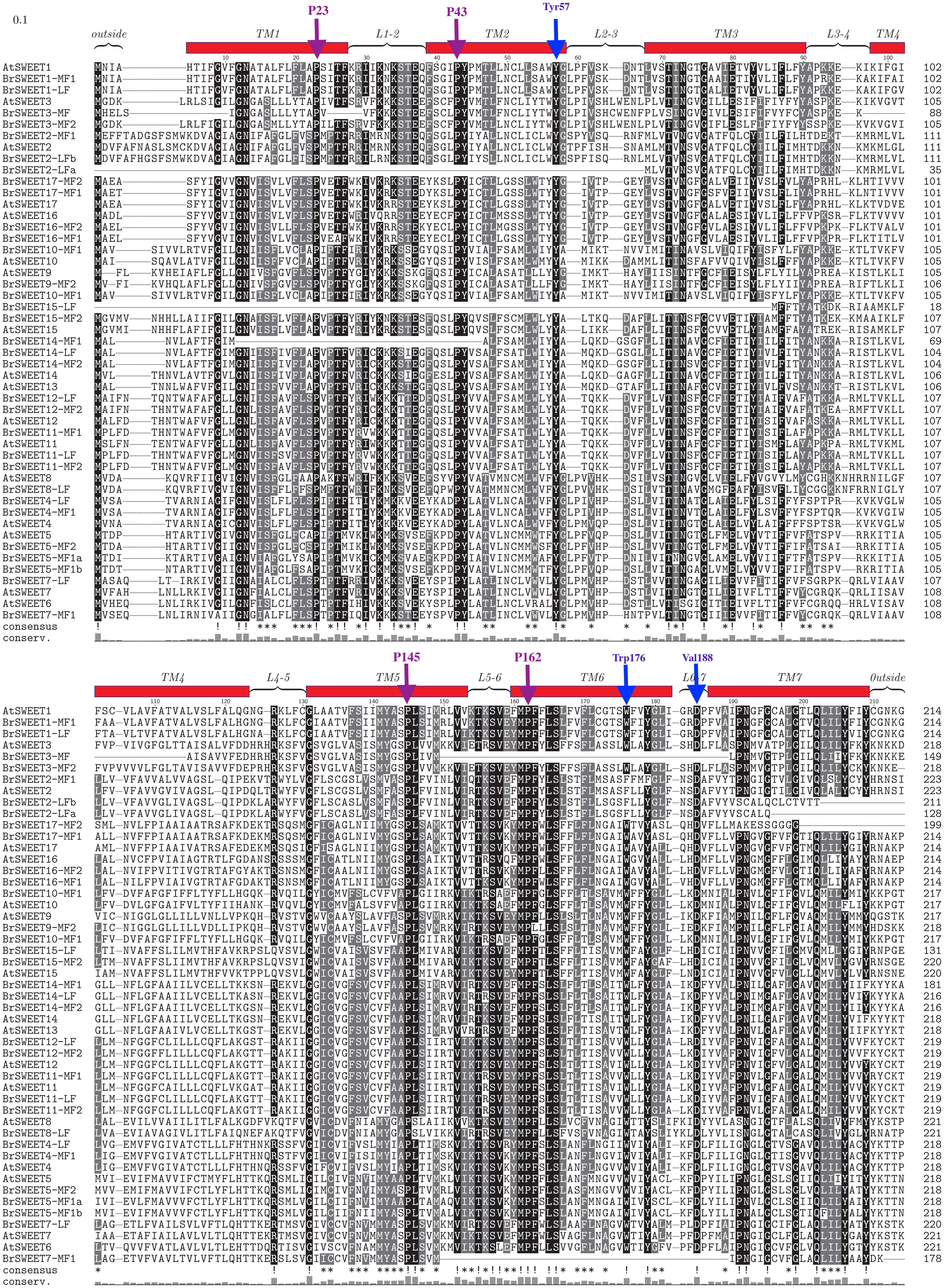
0.1
outside
TM1
L1-2
TM2
L2-3
TM3
L3-4
TM4
P23
P43
Tyr57
AtSWEET1
BrSWEET1-MF1
BrSWEET1-LF
AtSWEET3
BrSWEET3-MF
BrSWEET3-MF2
BrSWEET2-MF1
AtSWEET2
BrSWEET2-LFb
BrSWEET2-LFa
BrSWEET17-MF2
BrSWEET17-MF1
AtSWEET17
AtSWEET16
BrSWEET16-MF2
BrSWEET16-MF1
BrSWEET10-MF1
AtSWEET10
AtSWEET9
BrSWEET9-MF2
BrSWEET10-MF1
BrSWEET15-LF
BrSWEET15-MF2
AtSWEET15
BrSWEET14-MF1
BrSWEET14-LF
BrSWEET14-MF2
AtSWEET14
AtSWEET13
BrSWEET12-LF
BrSWEET12-MF2
AtSWEET12
BrSWEET11-MF1
AtSWEET11
BrSWEET11-LF
BrSWEET11-MF2
AtSWEET8
BrSWEET8-LF
BrSWEET4-LF
BrSWEET4-MF1
AtSWEET4
AtSWEET5
BrSWEET5-MF2
BrSWEET5-MF1a
BrSWEET5-MF1b
BrSWEET7-LF
AtSWEET7
AtSWEET6
BrSWEET7-MF1
consensus
conserv.
TM4
L4-5
TM5
L5-6
TM6
L6-7
TM7
Outside
P145
P162
Trp176
Val188

Outside

```
                 220           230                          240
AtSWEET1         -EKSA-D-AQKD-E-KSVEMKDDE-KKQNVVNG-K-QDLQV                              247
BrSWEET1-MF1     -EKSTD-D-AEKD-EKKTVEMKDEE-KKKQNVVNG-K-KQEQQV                           251
BrSWEET1-LF      -EKS-AEKD-D-KSLEMKGEE-KKQNVVNG-K-QQEQQV                                246
AtSWEET3         LAPTTMVITKRNDHDDKN-K-ATLEFVVDV-DRNSDTNE-KNS-NNASSI                     263
BrSWEET3-MF      -APITTTVVGKWD-HEKN-K-SKLKLVVDI-DHDADADD-NEK-KFMNAC                     192
BrSWEET3-MF2     -TPIITTVMSKWD-DEKN-K-RELELVVDV-DHDGHAKE-K-KFNYAC                       259
BrSWEET2-MF1     -EEEA-KEP-LIVSYV                                                       236
AtSWEET2         -EEET-KEP-LIVSYV                                                       236
BrSWEET2-LFb     -TGVL                                                                  215
BrSWEET2-LFa     -                                                                      128
BrSWEET17-MF2    -GEAE-QEEE-E-RWRKRDDKDTKGRT-G-CGAGWEKTTHA-AEKIRFRFIF                   244
BrSWEET17-MF1    -AGLSNG-SSEIA-ADEE-E-GLTSRA-PLLS                                       240
AtSWEET17        -VGLSNG-LSEIA-QDEE-E-GLTSRVE-PLLS                                      241
AtSWEET16        -I-VEDE-E-GLIPNQ-PLLA                                                  230
BrSWEET16-MF2    -N-I-VDEE-E-ALAPSQ-PLLS                                                231
BrSWEET16-MF1    -N-V-KDEE-E-ALAPSQ-PLLS                                                231
BrSWEET10-MF1    -KV-L-EPPVINL-Q-EISDHVVDVVRLSS-MVCSSQM-RTLV-PQDSADMEDTIT-IDEKIKGDIE-KMKES-KEM-LLISKN   289
AtSWEET10        -KV-L-EPPGIKL-Q-DISEHVVDVVRLST-MVCNSQM-RTLV-PQDSADMEATID-IDEKIKGDIE-KNKDE-KEV-FLISKN   289
AtSWEET9         -TDLP-T-ENQLANK-T-DVNEVPIVAVELPD-V-GSDNVEGSVR-P-M-K                    258
BrSWEET9-MF2     -TDLPKLTS-T-ENQPTNI-T-NLNEVAIVAVELSD-A-RAENVEGSVR-P-MTP-NSSTTA         270
BrSWEET10-MF1    -KV-L-EPPVINL-Q-EISDHVVDVVRLSS-MVCSSQM-RTLV-PQDSADMEDTIT-IDEKIKGDIE-KMKES-KEM-LLISKN   289
BrSWEET15-LF     -KLD-T-EKKMNPS-D-QQLKSVVVMSPLGV-SEVHPIDVNVT-EPVD-PFSDAVQHKDPSKVTKEKEPATD-DGKCH-VET-ARHESV   209
BrSWEET15-MF2    -KLD-I-GKKNNSS-S-EQLKTIVVMSPLGL-SEMHPVDVTVT-EPVI-PLSYTVHHEDPSKITKEEETSTE-AAQSH-VE-TAPSRI   297
AtSWEET15        -K-PEKINSS-E-QQLKSIVVMSPLGV-SEVHPV-VT-ESVD-PLSEAVHHEDLSKVTKVEEPSIE-NGKCY-VEA-TRPETV   292
BrSWEET14-MF1    -P-K-VDDTEKP-K-TVQDHSIDMVKLTSTTPVSGD-MTVH-PQTH-AGDLEGQME-KKV-TNQIQT    238
BrSWEET14-LF     -P-K-TDDTEKP-K-TVAVHSIDMFKLAS-TPVSSE-LTVH-PQTH-GGGDLEGQME-KKV-ANQIQT   273
BrSWEET14-MF2    -P-K-TDDTEKP-K-TVSGHSIDMVKLAS-TPASGD-LKAP-PQTH-GGDLEGQIE-KEM-ANQIQT    272
AtSWEET14        -PL-V-VDETEKP-K-TVSDHSINMVKLSS-TPASGD-LTVQ-PQTNPDVSHPIKTHGGDLEDQMD-KKM-PN-   281
AtSWEET13        -P-V-AQKTDKS-K-DVSDHSIDIAKLTT-VIPGAV-LDSAVHQPPALHNVPETKIQLTEVKSQNMTDPKDQIN-KDV-QKQSQV   294
BrSWEET12-LF     -PSD-L-VEKELEA-A-KLPEVSIDMLKLGT-L-ASPEPAEITVV-PTANKCIC-NDRKAETENGQGVKNG-T-HSTAAC   288
BrSWEET12-MF2    -PSD-L-VEKELEA-A-KLPEVSIDMLKLGT-LVASPEPAVITVV-RPVSMCVC-NDKKAEAGNG-A    277
AtSWEET12        -PSD-L-VEKELEA-A-KLPEVSIDMVKLGT-L-TSPEPVAITVV-RSVNTCNC-NDRNAEIENGQGVRN-SAATT   285
BrSWEET11-MF1    -PPQ-L-GEKEVEA-A-KLPEVSLDMLKLGT-V-SSPEP-IAVV-RQTNKCTC-GNDQRSEAELMDKTASSPLPQ-HLHEHE   290
AtSWEET11        -SPH-L-GEKEVEA-A-KLPEVSLDMLKLGT-V-SSPEP-ISVV-RQANKCTC-GNDRRAEIEDGQTPKHG-KQS-SSAAAT   289
BrSWEET11-LF     -PPH-L-EEKEVEA-A-KLPEVTLDMLKLGT-V-SSPET-ITAV-RQANKCTC-GNDRRAEIEDGENAKNG-KQS-SSATAT   289
BrSWEET11-MF2    -PPH-L-GEKEVEA-A-KLPEVSLDILKLGT-V-SSPE-V-RQANKCTC-GNDRRPEVEDGQNAKNG-KQS-SSASPT   285
AtSWEET8         -KE-K-TVKPSEVEI-SATERV                                                 239
BrSWEET8-LF      -RDE-D-KTKPSEIEI-PATA                                                  238
BrSWEET4-LF      -KDD-EEE-D-NLSKANSQL-QLSG-NQGQAKGVPA                                   248
BrSWEET4-MF1     -KD-EEE-D-NLSKAN-L-QLSG-NEEHAKRVSA                                     245
AtSWEET4         -KDDE-D-EEDE-E-NLSKVNSQL-QLSG-NSGQAKRVSA                               251
AtSWEET5         -WNDD-D-EDKE-K-RYSNAGIE-LGQA                                           240
BrSWEET5-MF2     -WNDE-D-GDKE-K-RFTNAEIQ-LDRA                                           240
BrSWEET5-MF1a    -WKDD-D-EDNE-N-SNSNAEIE-HSQA                                           240
BrSWEET5-MF1b    -WSDD-D-EDKE-K-SNLNAEIE-LSQA                                           240
BrSWEET7-LF      -KMMA-E-RQPM-I-GLSSVVVR-IGSEKVA-QPSA                                   249
AtSWEET7         -RIMA-E-RENQPGY-V-GLSSAIAR-TGSEKTANT-NQE-PNNV                          258
AtSWEET6         -GIME-E-RKNRLGYVGEV-GLSNAIAQ-TEPENIPYL-NKR-VSGV                        261
BrSWEET7-MF1     -                                                                      178
consensus
conserv.
```

- X non conserved
- X ≥50% conserved
- X ≥80% conserved
