# Supplementary figures and images for: The Expanded SWEET Gene Family Following Whole Genome Triplication in Brassica rapa
Source: Genes (Basel). 2019 Sep 18;10(9):722. doi: 10.3390/genes10090722 (PMC6771021; doi:10.3390/genes10090722)

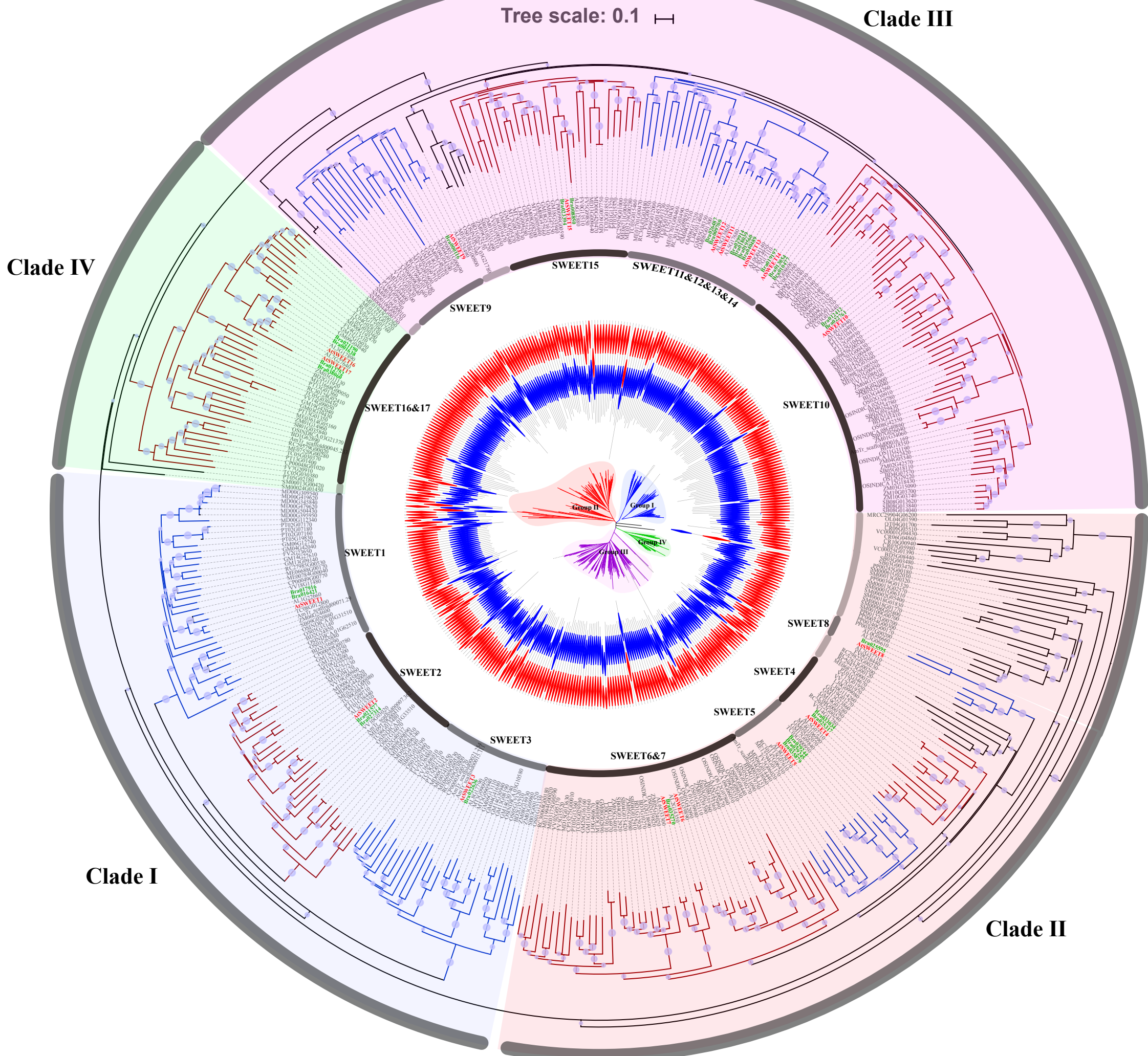

Supplement: Supplementary file 1 [file genes-10-00722-s001.zip › Supplementary files/Figure S1.pdf]
